# 超高效液相色谱-静电场轨道阱高分辨质谱快速筛查和确证凉茶中167种非法添加药物

**DOI:** 10.3724/SP.J.1123.2021.07006

**Published:** 2022-03-08

**Authors:** Jiawen HE, Jiaxin WEN, Yaxiong LIU, Jiazhe HU, Yajing CAO, Yuhong LAI

**Affiliations:** 广东省药品检验所, 广东 广州 510663; Guangdong Institute for Drug Control, Guangzhou 510663, China; 广东省药品检验所, 广东 广州 510663; Guangdong Institute for Drug Control, Guangzhou 510663, China; 广东省药品检验所, 广东 广州 510663; Guangdong Institute for Drug Control, Guangzhou 510663, China; 广东省药品检验所, 广东 广州 510663; Guangdong Institute for Drug Control, Guangzhou 510663, China; 广东省药品检验所, 广东 广州 510663; Guangdong Institute for Drug Control, Guangzhou 510663, China; 广东省药品检验所, 广东 广州 510663; Guangdong Institute for Drug Control, Guangzhou 510663, China

**Keywords:** 超高效液相色谱, 静电场轨道阱高分辨质谱, 非法添加药物, 凉茶, ultra high performance liquid chromatography (UHPLC), electrostatic field orbitrap high-resolution mass spectrometry (Orbitrap HRMS), illegally added medicines, herbal tea

## Abstract

基于质谱数据库,建立了超高效液相色谱-静电场轨道阱高分辨质谱(UHPLC-Orbitrap HRMS)快速筛查和确证凉茶中167种非法添加药物的方法。通过调研,选定了解热镇痛药、糖皮质激素、抗菌药、抗组胺药等167种药物,并利用Orbitrap HRMS和TraceFinder软件采集和记录每种药物的信息,建立高分辨质谱数据库。样品以50%(v/v)甲醇水溶液超声提取。样品溶液采用Waters XBrigde BEH C18色谱柱(100 mm×2.1 mm, 2.5 μm)分离,0.1%(v/v)甲酸水溶液-0.1%(v/v)甲酸乙腈溶液梯度洗脱。Orbitrap HRMS以全扫描/数据依赖二级质谱扫描(Full MS/dd-MS^2^)模式,正、负离子同时切换采集数据。将样品数据导入TraceFinder软件,以母离子精确质量数、保留时间、碎片离子精确质量数等进行数据库自动筛查。若某成分的母离子精确质量数实测值与理论值偏差小于5×10^-6^,保留时间偏差小于20 s,至少一个碎片离子精确质量数实测值与理论值偏差小于5×10^-6^,且二级质谱与数据库收录谱图相似,可判定该成分检出。结果表明,方法特异性好,各成分线性关系良好,相关系数(*r*)大于0.99。培氟沙星等5种成分存在本底干扰,不宜采用该法进行定量,其余162种成分回收率为66.4%~118.1%,精密度RSD (*n*=6)为0.1%~16.1%。方法在0.2 mg/kg下可筛查83种成分,1.0 mg/kg下可筛查167种成分。方法应用于245批样品,检出12批问题样品,阳性成分有对乙酰氨基酚、双氯芬酸钠、氯苯那敏等,并检出标准方法外成分金刚烷胺、右美沙芬、溴苯那敏和环丙沙星。方法检测速度快,分析成分多,筛查结果准确,为凉茶非法添加药物高通量筛查提供了新的技术支撑。

## Abstract (en)

An accurate mass database and a method based on ultra high performance liquid chromatography-electrostatic field orbitrap high resolution mass spectrometry (UHPLC-Orbitrap HRMS) were developed. These were applied in the screening and identification of illegally added medicines in herbal tea. Based on investigations, 167 medicines were selected to build an accurate MS database; these medicines included antipyretic analgesics, glucocorticoids, antibiotics, and antihistamines, among other categories. The database was established using Orbitrap HRMS and TraceFinder software. The database carried information on all selected compounds, including the molecular formula, accurate mass of precursor ions and fragment ions, retention time, and mass spectra. The samples were ultrasonically extracted with a 50% (v/v) methanol aqueous solution. The extracted solutions were separated using a Waters XBrigde BEH C18 column (100 mm×2.1 mm, 2.5 μm). As the mobile phases, 0.1% (v/v) formic acid aqueous solution and acetonitrile containing 0.1% (v/v) formic acid were used, with gradient elution. The sample solutions were analyzed by Orbitrap HRMS in the full-scan MS and data-dependent MS/MS acquisition modes (Full MS/dd-MS^2^). Positive and negative polarity data were simultaneously acquired. Some parameters were optimized to increase the peak intensity and sensitivity of all compounds. The resolutions in the full-MS scan and dd-MS^2^ scan were set to 70000 and 17500, respectively. In the full-MS mode, scanning was performed in the range of *m/z* 100 to 1000. In the MS/MS mode, the normalized collision energy (NEC) was set to 20%, 40%, and 60% for each compound. The inclusion list was not used during the measurement, and the dynamic exclusion time was set to 10.0 s. The loop count was set to 5. After acquiring the sample data with these conditions using Orbitrap MS, they were imported into TraceFinder software, through which the sample information was extracted and automatically matched with the information on compounds in the MS database. Screening and identification were conducted by comparing the retention times as well as the exact masses of precursor ions and fragment ions that were experimentally measured. If the errors between the experimentally and theoretically obtained masses of the precursor ions were below 5×10^-6^ and the deviations in retention times were less than 20 s, then suspicious positive compounds might be identified. Furthermore, if such compounds possess more than one similar fragment ion with a mass tolerance below 5×10^-6^, and exhibit similar ion distributions in the MS/MS profiles (compared to those in the database), they could be confirmed to be the same. The validation result showed that all compounds had good linear relationships, with correlation coefficients (*r*) greater than 0.99. Because pefloxacin, norfloxacin, desloratadine, astemizole and clindamycin had background interference, the method was not suitable for their quantification. Following experiments using three spiked concentrations, the recoveries of the rest 162 compounds were found to be in the range of 66.4%-118.1%, and the relative standard deviations (RSDs, *n*=6), in the range of 0.1%-16.1%. When the limit of detection (LOD) was 0.2 mg/kg, 83 compounds were detected, while when the LOD was 1.0 mg/kg, 167 compounds were detected. All compounds were matched successfully to the standard added sample with the MS database in TraceFinder software. To lower the likelihood of false positive and false negative results, a quality control method was recommended. The method was applied to analyze 245 herbal tea samples, among which 12 positive samples were detected. Thirteen positive compounds were found, including acetaminophen, diclofenac sodium, chlorpheniramine, brompheniramine, dexamethasone, dexamethasone 21-acetate, prednisone, prednisone 21-acetate, metronidazole, erythromycin, ciprofloxacin, amantadine, and dextromethorphan. In particular, amantadine, dextromethorphan, brompheniramine, and ciprofloxacin were newly detected, compared to standard methods. The developed method is rapid and accurate, and will be useful in the high-throughput screening of illegally added medicines in herbal tea.

凉茶是两广地区流行的传统饮料,一般认为具有清热解毒、祛湿消滞的功效。有不法商家受利益驱使,在凉茶中非法添加感冒药、糖皮质激素等以增强宣称的功效,这种行为具有潜在的食品安全风险,并严重影响市场秩序。若误服含有解热镇痛药的凉茶,可能掩盖发热、咽痛等症状,延误疾病治疗。另一方面,随着监管力度加大,凉茶非法添加药物的种类不断增加,不限于已知的以对乙酰氨基酚为代表的解热镇痛药,涉及抗组胺药、抗生素、平喘药、镇咳药等,具有多样性、复杂性和未知性。

目前,凉茶非法添加药物的检测方法有薄层色谱法^[1]^、酶联免疫法^[2]^、高效液相色谱法^[1,3,4]^、液相色谱-质谱联用法^[5,6,7,8,9]^等。薄层色谱法、酶联免疫法、液相色谱法灵敏度较低,检测成分较少。液相色谱-三重四极杆质谱法是非法添加检测的经典方法,被BJS 201713等现行补充检验方法采用。此类方法一般利用多反应离子监测模式进行靶向检测,在新成分或未知成分识别上有一定的局限性。孙树周等^[5]^发现的茶碱、喷托维林和胡佳哲等^[6]^发现的甲硝唑、红霉素是补充检验方法外成分,超出标准范围,在标准检验中不能被有效识别。此外,若同时检测上述多种药物,需采用多个方法,检验效率低。因此,三重四极杆质谱的靶向检测模式已不能满足新形势下凉茶非法添加药物的检验需求,亟须建立一个覆盖药物尽量多的跨类别多成分高通量筛查方法。

静电场轨道阱高分辨质谱(Orbitrap HRMS)是近年研究热点,具有高分辨率、高质量精度优势,采集的质谱信息丰富,可实现上百种药物同时检测和非靶向未知物筛查,在农兽药残留、环境污染物、化妆品致敏物质高通量筛查中已有较成熟的研究^[10,11,12,13,14,15,16,17,18]^,但对凉茶非法添加药物筛查的研究较少。刘桂联等^[9]^利用Orbitrap HRMS快速筛查和确证凉茶中56种降脂、降压类药物,给出了示范性研究。然而非法添加药物远多于56种,现有研究仍未覆盖凉茶高风险添加药物,未发挥高分辨质谱的明显优势。

参考其他领域的研究经验,结合质谱数据库可以极大限度发挥高分辨质谱在高通量分析中的优势。本文通过调研,针对性地选定解热镇痛、抗组胺、抗菌、消炎、减肥等167种非法添加高风险药物,建立高分辨质谱数据库。优化样品前处理、色谱-质谱条件,建立超高效液相色谱-静电场轨道阱高分辨质谱快速筛查和确证凉茶中167种药物的方法,实现了非法添加药物的高通量、高精度筛查,突破当前检测局限,为打击凉茶非法添加提供新的技术支撑。

## 1 仪器与方法

### 1.1 仪器与试剂

超高效液相色谱仪VANQUIVE Optiplex、四极杆/静电场轨道阱高分辨质谱Q Exactive Orbitrap和TraceFinder 4.1软件(美国Thermo Scientific公司);分析天平MS205DU(瑞士Mettler公司);超纯水发生器Mili-Q Advantage A10(美国Millipore公司);超声波清洗器(昆山市超声仪器有限公司)。

乙腈、甲醇(色谱纯)购自美国Honeywell公司,甲酸、甲酸铵、乙酸铵、冰醋酸(色谱纯)购自上海麦克林生化科技有限公司。167种药物标准品包括解热镇痛药22种(详见表1中序号1~22)、抗菌药物47种(23~69)、激素41种(70~110)、抗组胺药物15种(111~125)、镇咳平喘药14种(126~139)、消化系统用药13种(140~152)、减肥药7种(153~159)和其他类8种(160~167),分别购自中国食品药品检定研究院、德国Dr. Ehrenstorfer公司和上海阿拉丁生化科技股份有限公司。

**表1:** 167种药物的分子式、母离子、保留时间、碎片离子和检出限

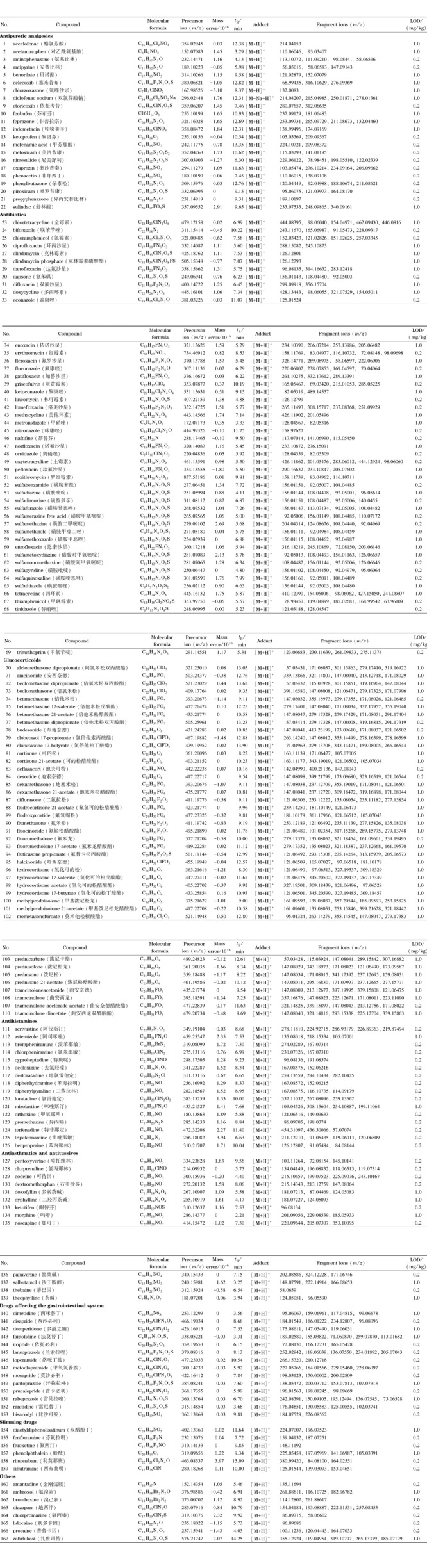

245批凉茶样品购自广东省内凉茶店,均为液体凉茶。

### 1.2 标准溶液配制

分别准确称取标准品10 mg,用甲醇溶解并定容至10 mL,不溶的标准品用冰醋酸或乙腈溶解后再用甲醇定容,配制成单标准储备液,于-20 ℃冰箱保存。

取适量各单标准储备液,用50%(v/v)甲醇水溶液配制成质量浓度为100 ng/mL的数据库采集用标准溶液,现用现配。

分别取适量对乙酰氨基酚、土霉素、环丙沙星、磺胺间甲氧嘧啶、氯霉素、兰索拉唑、红霉素、地塞米松单标准储备液,用50%(v/v)甲醇水溶液配制成质量浓度为10 ng/mL和50 ng/mL的混合标准质控溶液,现用现配。

### 1.3 样品溶液制备

取样前摇匀样品,称取1 g,置于20 mL容量瓶中,加入适量50%(v/v)甲醇水溶液,超声提取15 min。取出放冷,用50%(v/v)甲醇水溶液定容至20 mL, 0.22 μm滤膜过滤,待测。

### 1.4 分析条件

色谱柱:Waters XBridge BEH C18柱(100 mm×2.1 mm, 2.5 μm),柱温40 ℃,流动相A 0.1%(v/v)甲酸水溶液,流动相B 0.1%(v/v)甲酸乙腈溶液,流速0.3 mL/min。梯度洗脱程序:0~1.0 min, 5%B; 1.0~17.0 min, 5%B~95%B; 17.0~20.0 min, 95%B; 20.0~20.5 min, 95%B~5%B; 20.5~25.0 min, 5%B。进样量5 μL。

喷雾电压3.50 kV(正离子)和-3.20 kV(负离子),毛细管温度320 ℃;管状透镜电压50.0 V,辅助气加热温度350 ℃,鞘气流速4.55 L/min,辅助气流速3 L/min。扫描类型为全扫描/数据依赖二级质谱扫描(Full MS/dd-MS^2^),正、负离子同时转换,关闭目标离子列表(inclusion)。Full MS参数:分辨率70000,自动增益控制目标离子数(AGC target)1.0×10^6^,最大离子注入时间(IT)100 ms,扫描范围*m/z* 100~1000。dd-MS^2^参数:分辨率17500, AGC target为2.0×10^5^, IT为80 ms,循环次数(Loop count)5,归一化碰撞能量(NEC)20%、40%和60%,开启动态排除10 s。

### 1.5 质谱数据库的建立

注入数据库采集用标准溶液,按仪器分析条件采集每个药物的质谱图,使用仪器工作站提取保留时间、母离子和碎片离子精确质量数等数据。将数据导入TraceFinder软件,编辑药物化学信息,建立167种药物的高分辨质谱数据库,主要信息见表1。

### 1.6 筛查确证方法

注入样品溶液和混合标准质控溶液,按仪器分析条件采集数据。将数据导入TraceFinder软件,与数据库中药物的保留时间、母离子和碎片离子精确质量数进行自动筛查匹配。当满足母离子精确质量数偏差小于5×10^-6^(5 ppm),保留时间偏差小于20 s,至少一个碎片离子精确质量数偏差小于5×10^-6^,可判断为检出。检出成分的二级质谱图与数据库收录的标准品谱图比对一致,可确证为同一物质。若检出地塞米松或倍他米松,需更换BJS 201713色谱条件进行区分。混合标准质控溶液为随行的质量监控,当检出限浓度的8种成分全部检出时,仪器灵敏度和质量轴稳定,筛查结果可信度高。

## 2 结果与讨论

### 2.1 数据库成分的选择

凉茶跨类别添加药物的问题非常突出,根据宣称的功效,可加入解热镇痛药、抗菌药物(抗生素)、糖皮质激素、抗组胺药物等,消化系统药物和减肥药也可能非法添加到凉茶中。综合考虑,本文针对性选择了167种药物建立高分辨质谱数据库,基本覆盖了常见阳性成分和非法添加高风险药物。伪麻黄碱和咖啡因是感冒复方制剂中常用的药物,也是植物中自然存在的生物碱,其检出难以判断是否非法添加,因此不纳入数据库中。

### 2.2 样品前处理的优化

考察了水、50%(v/v)甲醇水溶液、70%(v/v)甲醇水溶液和甲醇作为提取溶剂对167种药物的提取效果。结果表明,大部分脂溶性药物不适宜采用水提取,其他3种提取溶液提取效果相当,但甲醇的体积分数影响个别药物的响应和稳定性。保留时间较小的吗啡、对乙酰氨基酚在纯甲醇中溶剂效应明显,峰形、响应较差。拉唑类成分不稳定,以兰索拉唑为例,24 h内在50%(v/v)甲醇水溶液、70%(v/v)甲醇水溶液和甲醇中分别降解15%、18%和23%,在50%(v/v)甲醇水溶液中稳定性较好。因此,50%(v/v)甲醇水溶液作为提取溶剂可以兼顾尽量多成分的响应和稳定性。

比较了不同体积提取溶剂对凉茶中目标成分的影响。1 g样品中分别加入50%(v/v)甲醇水溶液5、10、20和50 mL进行提取,对乙酰氨基酚保留时间较小,受基质干扰明显,其色谱表现能有效反映样品的干扰情况,故以对乙酰氨基酚为代表进行考察。结果显示,提取体积为5 mL和10 mL时,样品溶液颜色较深,离子流图基线噪声较大,干扰对乙酰氨基酚色谱峰;20 mL时,样品溶液颜色浅,离子流图基线相对平稳,对乙酰氨基酚色谱峰无明显干扰;50 mL时,样品溶液几乎无色,离子流图基线平稳,色谱峰无干扰。为获得更高的灵敏度,实验选择提取溶剂体积为20 mL。

考察了超声15 min、超声30 min或振摇15 min的提取效果,3种提取方式无差异。

综上,采用50%(v/v)甲醇水溶液20 mL超声15 min作为提取方法。

### 2.3 色谱条件优化

考察了药物在Waters XBridge BEH C18(100 mm×2.1 mm, 2.5 μm)、Thermo Hypersil GOLD(100 mm×2.1 mm, 1.9 μm)和Phenomenex Kinetex C18(100 mm×2.1 mm, 2.6 μm)3款色谱柱上的色谱行为。四环素类药物对色谱条件敏感,以土霉素为例进行说明。土霉素在Thermo Hypersil GOLD上柱拖尾明显(见图1a),在Phenomenex Kinetex C18柱和Waters XBridge BEH C18柱上峰形良好(见图1b和1c),后者峰宽更窄,峰形更尖锐。因此,选择整体表现更佳的Waters XBridge BEH C18柱作为方法推荐的色谱柱。

**图1:**
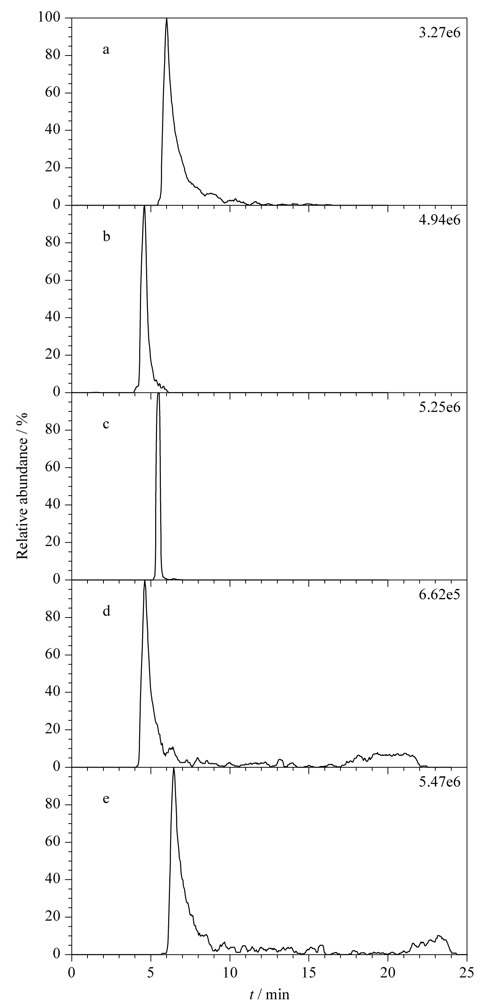
土霉素在不同色谱条件下的色谱图

比较了0.1%(v/v)甲酸水溶液-0.1%(v/v)甲酸乙腈、0.01 mol/L乙酸铵-乙腈和0.01 mol/L甲酸铵-乙腈作为流动相时药物的分离情况,继续以土霉素为例进行说明。结果表明,当流动相含甲酸时,土霉素峰形好、响应强(见图1b);但流动相含乙酸铵、甲酸铵时,土霉素色谱峰拖尾,响应减弱,灵敏度变差(见图1d和1e)。因此,选择0.1%(v/v)甲酸水溶液-0.1%(v/v)甲酸乙腈作为流动相体系。

### 2.4 质谱参数优化

本方法筛查的药物多达167个,优化时需保证每个药物被有效采集。Orbitrap质谱参数中分辨率和最大驻留时间的设置相对固定,其他可调参数有Top*N*、动态排除功能。Top*N*代表一级质谱响应前*N*强的离子进行二级质谱采集,由循环次数和每次二级质谱采集的离子数(MSX count)决定,计算公式为*N*=Loop count×MSK count。当MSK count默认为1, Loop count设置为5时,能获得数量较多、质量较好的二级图谱。动态排除是指强度占优势的离子做过二级质谱后将其排除,避免重复采集,以多获得不同质荷比离子的二级图谱。以本方法为例,对乙酰氨基酚、甲硝唑、氨基比林在凉茶中常见添加量为600~2000 mg/kg,茶碱、可待因常见添加量为20~60 mg/kg,剂量至少相差10倍。上述成分保留时间在3.3~4.4 min,开启动态排除前,对乙酰氨基酚等高添加量成分在响应上占有优势,抑制了茶碱、可待因的采集。开启后情况得到显著改善,解决了剂量差异的干扰问题。

此外,张蓉等^[10]^认为开启目标离子列表(inclusion)可以强制采集目标质量数,增强检测灵敏度。但考察发现,当列表化合物数量从50迅速增加到150时,二级质谱的采集点数锐减,部分图谱没有达到峰顶采集的要求,图谱质量不佳,影响数据库筛查的准确性。因此,方法关闭inclusion列表。

### 2.5 同分异构体的处理

167种药物中有8组同分异构体,分别是酮洛芬和芬布芬、磺胺间甲氧嘧啶和磺胺对甲氧嘧啶、赛庚啶和萘替芬、泼尼松龙和可的松、倍他米松和地塞米松、甲基泼尼松龙醋酸酯和地索奈德、倍他米松醋酸酯和地塞米松醋酸酯和曲安奈德、多西环素和四环素。除地塞米松和倍他米松外,其余7组的保留时间或碎片离子有区别,筛查时同分异构体间不会相互混淆。如酮洛芬(10.54 min,特征碎片离子*m/z* 105.03369、209.09567)和芬布芬(10.93 min,特征碎片离子*m/z* 237.09129、181.06483)的保留时间相近,但是特征离子不同。地塞米松和倍他米松为差向异构体,在本方法条件下保留时间均为9.11 min,拥有相同的碎片离子*m/z* 147.080、355.190、171.080、121.065,不能有效区分。经考察,更换BJS 201713的色谱条件能有效分离两种激素,可作为后续的确认手段。

### 2.6 方法学验证

2.6.1 特异性

取阴性样品溶液、加标溶液进样分析,记录离子流图和多级质谱图,进行数据库筛查。结果显示,阴性样品中无目标成分检出,加标样品中均筛查出目标成分,样品基质对筛查结果无干扰。

2.6.2 检出限

在阴性样品中加入适量标准溶液,制得质量浓度为10 ng/mL和50 ng/mL的加标溶液,从低至高进样确定各成分的检出限,其中10 ng/mL对应0.2 mg/kg的检出限水平,50 ng/mL对应1.0 mg/kg的检出限水平。汪洋等^[13]^根据欧盟EC/657/2002中质谱分析方法鉴定点数的要求,认为高分辨质谱定性至少需要母离子和一个子离子。

基于这一原则,本方法判断目标成分检出的参数指标为样品溶液中有与数据库中药物保留时间一致的色谱峰,并且母离子精确质量数误差小于5×10^-6^,至少有一个精确质量数误差小于5×10^-6^的碎片离子。当保留时间、母离子和碎片离子3项指标在数据库筛查均满足要求时,对应的最低浓度为检出限,结果见表1。本方法在0.2 mg/kg下可筛查出83种成分,1.0 mg/kg下可筛出167种成分,可满足凉茶中多种非法添加药物同时筛查的要求。

2.6.3 线性关系

分别配制质量浓度为10、20、30、50、80、100、200、300、500 ng/mL的系列标准溶液,考察各成分的线性关系。各成分按实际情况选取5个浓度点,以质量浓度为横坐标(*x*, ng/mL),以对应母离子提取离子流图中的色谱峰峰面积为纵坐标(*y*),绘制标准曲线,结果见附表1(详见 http://www.chrom-China.com)。结果表明,各成分线性关系良好,相关系数(*r*)大于0.99。

2.6.4 回收率和精密度

以1倍、2倍和10倍检出限浓度进行加标试验,采用单点法以母离子提取离子流图中的色谱峰峰面积计算回收率,每个浓度水平测定6次,计算RSD。由于定量分析时仅参考母离子提取离子流图,缺少碎片离子的特征性,培氟沙星、诺氟沙星、地氯雷他定、阿司咪唑和克林霉素受基质干扰存在本底值,因此不宜采用本方法对以上成分进行定量。结果显示,其余成分回收率为66.4%~118.1%, RSD为0.1%~16.1%(*n*=6),见附表1。将加标溶液进行数据库筛查,全部成分均匹配检出,准确率达到100%。

以质控溶液的8种代表成分考察基质效应。考察方法为分别配制溶剂标准曲线和基质曲线,以质量浓度和峰面积绘制标准曲线,按基质效应系数=溶剂标准曲线斜率/基质标准曲线斜率,计算基质效应系数。结果表明,8种成分的基质效应系数为0.91~1.14,说明无明显基质效应。

### 2.7 样品测定

2.7.1 筛查和确证结果

应用本方法对245批凉茶进行筛查,检出12批阳性样品,检出成分有对乙酰氨基酚、双氯芬酸钠、氯苯那敏、溴苯那敏、地塞米松醋酸酯、地塞米松、泼尼松醋酸酯、泼尼松、甲硝唑、红霉素、环丙沙星、金刚烷胺和右美沙芬。其中金刚烷胺、右美沙芬、溴苯那敏、环丙沙星是新发现的补充检验标准外成分。

以金刚烷胺为例简单说明本方法的筛查确证过程,阳性样品中对应的母离子提取离子流图、一级质谱图和二级质谱图见图2。

**图2:**
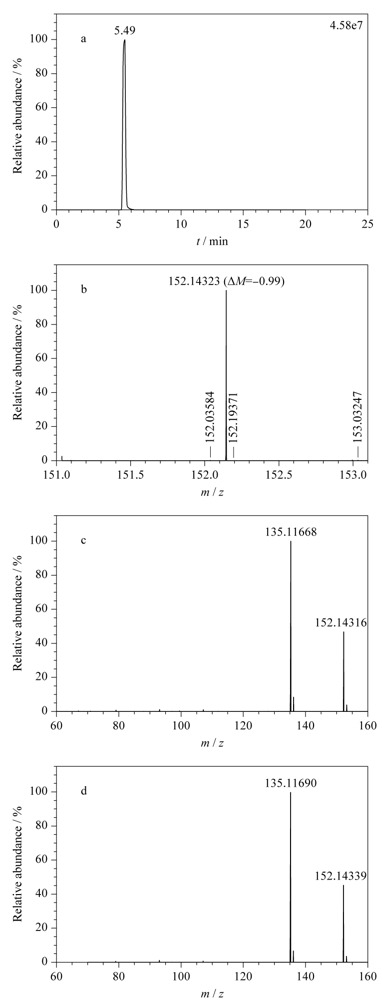
金刚烷胺阳性样品的色谱图和质谱图

数据库中,金刚烷胺的保留时间5.46 min,理论母离子*m/z* 152.14338,特征碎片离子*m/z* 135.11694。在母离子提取离子流图中可疑峰保留时间为5.49 min(见图2a),与数据库中收录的保留时间一致,满足保留时间要求。一级质谱图母离子精确质量数实测值为*m/z* 152.14323,与理论值相比质量偏差为-0.99×10^-6^(见图2b),小于5×10^-6^,符合母离子质量数要求。二级质谱图有一致的特征碎片离子(*m/z* 135.1169),且离子整体分布趋势一致(见图2c和2d),可确证为同一物质。

2.7.2 假阳性和假阴性分析

样品初筛有14批可疑样品,经进样序列分析、重新进样,有2批对乙酰氨基酚假阳性,是连续进样高浓度阳性样品,仪器污染导致。高浓度交叉污染是非法添加药物质谱分析中的常见问题,要求检验人员警惕高含量阳性样品后的弱阳性结果。

大批量分析时,污染积累会转化为持续的本底污染。如图3a所示,对乙酰氨基酚(母离子*m/z* 152.07061)持续污染后,其母离子提取离子流图基线整体上移至相对丰度的20%,灵敏度变低,并干扰了质量数相近的金刚烷胺(母离子*m/z* 152.14338)。当对乙酰氨基酚和金刚烷胺响应相当时,应产生*m/z* 152.07061通道和*m/z* 152.14338通道的提取质谱图;但当本底存在对乙酰氨基酚污染且金刚烷胺响应弱时,四极杆区分能力有限,仅获得*m/z* 152.07061通道的提取质谱图,导致金刚烷胺的二级质谱中出现对乙酰氨酚特征离子(*m/z* 110.06007)(对比图见图2c和3b),破坏碎片离子整体分布,造成误判。

**图3:**
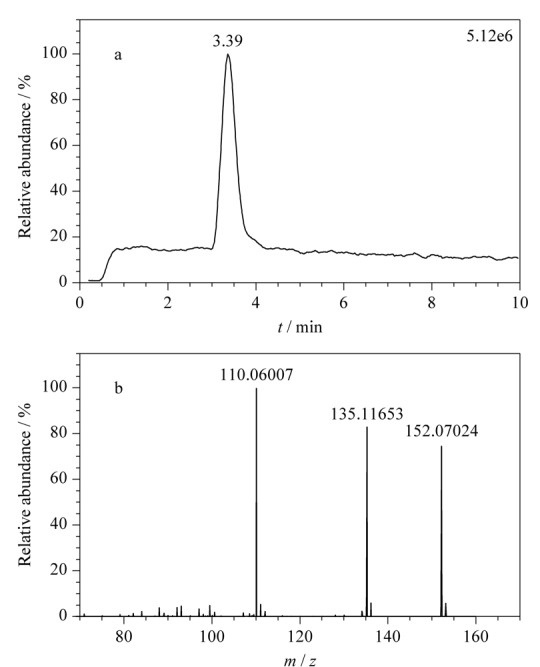
污染状态下(a)对乙酰氨基酚的色谱图和(b)金刚烷胺的质谱图

为方便检验人员及时发现问题,本方法引入标准质控溶液随行进样的手段,对分析过程进行质量控制。质控溶液设置为检出限浓度,包含了对乙酰氨基酚等8种正、负离子化合物,兼顾了常见的高浓度污染成分和不稳定成分,有一定的代表性。本方法建立的质谱数据库已录入药物信息,在无标准品情况下亦可以进行定性筛查。此时利用质控溶液可以监测灵敏度、质量轴稳定性和仪器状态,及时发现潜在的污染或降解,减少假阳性和假阴性,提高筛查结果的可信度。

## 3 结论

基于质谱数据库,本文建立了超高效液相色谱-静电场轨道阱高分辨质谱快速筛查和确证凉茶中167种非法添加药物的方法,可在1 h内完成样品前处理、检测和数据库筛查分析,检测效率高。方法应用于实际样品,有效检出多种阳性成分,筛查准确,靶向命中率高。本方法利用Orbitrap HRMS,以Full MS/dd-MS^2^模式采集的数据具有挖掘性,数据库中非法添加药物增加后,只需要将已有样品数据进行新一轮筛查,即可完成新增成分的分析,不需要重新处理样品或进行实验,灵活应对非法添加药物复杂多变的特点。本方法快速、准确,弥补了现时检测方法的不足,为打击凉茶非法添加药物行为提供了新的技术支撑。
